# Bone union and mobility outcomes for reconstructed open tibial fractures: a plastic surgical experience from a major trauma center

**DOI:** 10.3389/fsurg.2024.1348991

**Published:** 2024-02-01

**Authors:** Sadhishaan Sreedharan, Frank Bruscino-Raiola, Philip Lew, Yuan Ling, Scott Ferris

**Affiliations:** ^1^ Plastic, Hand and Faciomaxillary Surgery, The Alfred, Melbourne, VIC, Australia; ^2^ Department of Radiology, The Alfred, Melbourne, VIC, Australia

**Keywords:** open tibial fracture, microsurgery, lower limb reconstruction, trauma, reconstruction

## Abstract

**Introduction:**

The goal in open tibial fracture management is to achieve a united tibia in an extremity that allows pain free mobilization. The objective of this study was to assess factors that lead to this functional outcome in lower limb reconstruction, from a plastic surgical perspective.

**Materials and methods:**

The Plastic and Reconstructive Surgery lower limb database at a tertiary trauma hospital was searched for open tibial injuries from February 2015 to March 2020. The nature and severity of injury, timing and details of all operations including reconstructions were collected prospectively. Mobility including gait aids, pain, and complications were retrospectively collected. Union was assessed in two ways, depending on fracture location. Metaphyseal and diaphyseal tibial fractures were provided mRUST scores (union defined as RUST > 13) and epiphyseal tibial fractures were categorically classified as “united” or “non-union” by two independent radiologists.

**Results:**

During the five-year study period there were 148 open leg injuries in the database. Twenty-one patients underwent a primary amputation due to severity of their initial injury. One hundred patients underwent primary limb salvage. Sixty-one patients in the limb salvage group achieved primary tibial union with a mean follow-up time of 19.4 months post injury. Twenty-three additional patients were confirmed to subsequently unite. Patient who achieved union were more likely to mobilise without gait aids.

**Discussion:**

In this study definitive external fixation and soft tissue infection were both associated with higher rates of non-union. Longer times to soft tissue reconstruction was not associated with an increase in acute soft tissue complications. More importantly bone union, pain and mobility did not decline. After undertaking a primary limb salvage pathway for 100 patients, the ultimate tibial fracture union rate was 84% and the confirmed ambulation rate was 96%.

## 1 Introduction

Open lower limb fractures typically arise from high energy injuries, resulting in soft tissue defects overlying exposed tibial fractures, often with periosteal stripping and contamination (1–3). Open tibial fractures are commonly associated with multi-traumas and are well known to lead to significant morbidity and mortality (1, 2, 4, 5). Management of these injuries is a logistical challenge and requires the co-ordination of multiple specialties including: the trauma unit, orthopedic surgery, plastic surgery, and intensive care (1, 2, 5). Successful limb salvage relies on the cornerstone principles of optimal resuscitation, early antibiotic administration, timely and complete debridements, fracture stabilization and appropriate soft tissue reconstruction (1–8).

Godina's landmark study in 1986 (9) created the principle of early soft tissue coverage. This was based on a lower rates of flap failure, post-operative infection, and hospital length of stay if flap coverage was performed within 72 h of injury (8, 9). With the advent of negative pressure wound therapy as a temporizing measure and antibiotic prophylaxis, the management of complex wounds was further transformed (3–5). Subsequent studies investigating the timing of soft tissue coverage in open lower limb fractures have advocated expanding the “early period” to two weeks (4, 5, 7, 10).

Current guidelines (3) advice for definitive soft tissue coverage within seven days of injury. Many of the studies upon which these guidelines are made focused on free flap failure and acute infections rates (1, 4, 7, 9). A vascularized free flap is, however, not the final outcome to measure success in lower limb salvage, but rather one important component of care. Success in lower limb salvage should be and has been defined as a pain-free extremity that allows independent mobility (5, 8). The primary aim of this study was to assess bone union, pain and mobilization for open tibial fractures referred to plastic surgery.

## 2 Materials and methods

This observational study was conducted using the lower limb database contemporaneously maintained by the Plastic, Hand and Faciomaxillary Surgery Unit at the Alfred Hospital, Victoria. This tertiary trauma center is the largest and busiest Trauma Service in Australasia (11). The lower limb database includes all open lower limb injuries referred to the Plastic and Reconstructive Surgery service. Ethics approval was obtained through the Alfred's ethics committee (665/19). Patients were included if they sustained an open tibial fracture between February 2015 and March 2020. Patients were excluded if their primary injury was closed, or if they were first referred for a secondary soft tissue reconstruction of an old, previously managed fracture.

The lower limb database prospectively collects patient's demographics, co-morbidities, mechanism of injury, injury severity and location, Gustilo-Anderson classification, mangled extremity severity score (MESS), timing of referral to plastic surgery, timing of all debridements, type and timing of fixation, and type and timing of soft tissue reconstruction. All early complications were collected and included in data collection during the patient's acute admission and early postoperative reviews. Later postoperative complications, mobility, and pain were assessed using subsequent patient medical records. Patients' mobility and pain data was collected from their most recent surgical outpatient notes, pain service reviews and physiotherapy assessments. Mobility was categorized as wheelchair bound; mobilizing with a prosthesis for amputees; mobilizing with the use of a gait aid; and independent mobility if no aids were required. Pain was categorized as: “no pain” if the patient reported no pain with mobilizing; “minor pain” if the patient reported pain but it did not impact function; and “chronic pain” if patient's pain was persistently affecting mobilizing and was referred to the pain service.

Clinical fracture union was collected from orthopedics outpatient notes in the patients' medical records, and additionally radiologic union for all cases was assessed using plain tibial radiographs reviewed by two independent consultant radiologists. For metaphyseal and diaphyseal tibial fractures the modified Radiographic Union Scale for Tibia (mRUST) score developed by Litrenta et al. in 2015 (12) was used. mRUST score is calculated by scoring each cortex on the anteroposterior and lateral views as: 1 = no callus; 2 = callus present; 3 = bridging callus; and 4 = remodeled, fracture not visible (score range 4–16) (12, 13). Definitive union has been determined as a mRUST score of 13 or greater (12, 13). If there was a discrepancy between the two radiologists, an average of the two mRUST scores was used. Epiphyseal tibial fractures, such as tibial plafond or plateau fractures, where the “four cortices” on radiographs were not involved, were categorically reported as “united” or “non-union”. Soft tissue infection was defined by combinations of clinical appearance (i.e., cellulitis, discharge, pus, and wound dehiscence) and positive wound culture.

All data collected was de-identified and analysed using Microsoft Excel software version 16.66.1 (Microsoft Corporation, Redmond, Washington, USA). Continuous data are expressed as median ± standard deviations (SD) and categorical data are expressed as counts and percentages. Statistical significance was set at a two-tailed, *p*-value of 0.05 and calculated using a Mann–Whitney *U* test for continuous data and a Fisher-exact test for categorical data. Post-hoc power calculations were done using dichotomous endpoints and a confidence interval of 95%. Statistical analysis was conducted with GraphPad Prism 9 software version 9.4.0 (GraphPad Software, San Diego, California, USA).

## 3 Results

**Figure 1:**
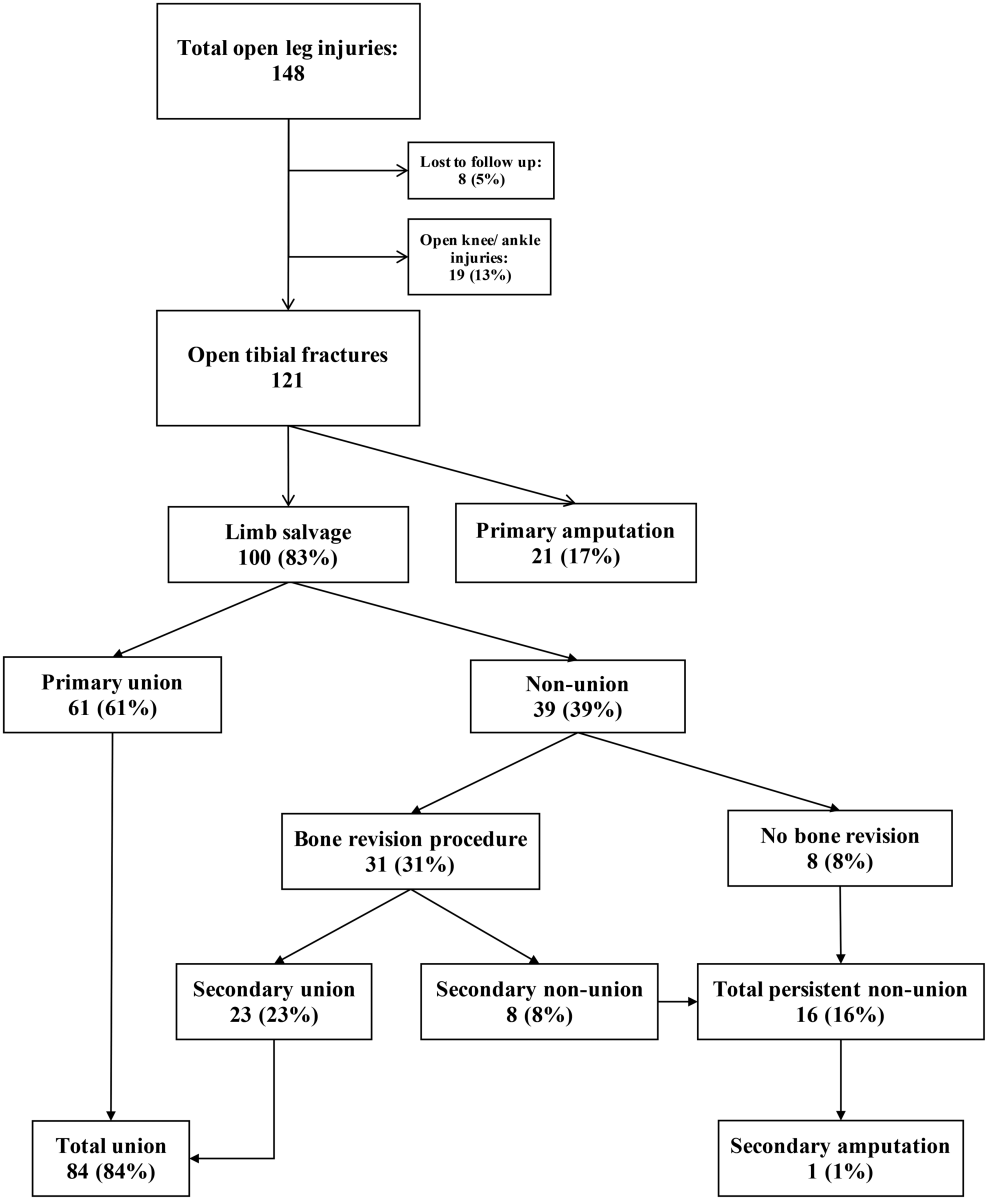
Flowchart of tibial union rates in all open tibial fractures that met study criteria.

A total of 148 open leg injuries were recorded in the database during the five-year study period. Of these, eight limbs were lost to follow up as their care was furthered elsewhere including interstate. Nineteen limbs were open ankle or knee joint injuries and did not actually have a tibial fracture to unite. There were 121 open tibial fractures that met the selection criteria (Figure 1). Of the 121 limbs, 100 limbs (83%) underwent primary limb salvage, and 21 limbs (17%) underwent a primary amputation. Of the 21 primary amputations, 8 were traumatic amputations at the scene and the remaining 13 were planned primary surgical amputation due to the severity of the soft tissue and skeletal injuries. The average follow-up time in the primary limb salvage group was 20.8 months ± 13.7 (SD).

There were 52 limbs that had a mRUST score ≥ 13 and nine epiphyseal fractures reported as “united” thus a total of 61 (61%) limbs that achieved primary bony union (Table 1). All 61 patients assessed as having achieved primary radiological bony union, also had an orthopedic clinic note stating that they had achieved clinical tibial union. Of the 39 (39%) limbs that did not achieve primary bony union, 31 underwent a further procedure for their non-union. Twenty-three of these limbs subsequently achieved secondary bony union by their last follow-up appointment. There were 84 (84%) limbs in total that achieved union and 16 (16%) limbs that had persistent non-union, giving a post-hoc power of 100%. Of the remaining 16 non-united patients (16%, 8 primary non-union and 8 persistent non-union), 15 patients were given more time to achieve union at their most recent appointment (Figure 1). Of these 16 non-united patients, four patients were ambulating pain free without gait aids despite their persistent non-union. Only one of the 100 (1%) primarily salvaged limbs went onto a secondary amputation for an infective non-union of the tibia.

**Table 1:** Summary of primary radiological union rates in patient with open tibial fractures that underwent limb salvage.

	Primary union	Non-union
Metaphysis & Diaphysis fractures	(mRUST ≥ 13)	(mRUST < 13)
Limbs	52	35
Epiphyseal fractures	“United”	“Non-union”
Limbs	9	4
Average time since injury (months ± SD)	19.4 ± 14.4	23.0 ± 12.5
TOTAL	61 (61%)	39 (39%)

SD, standard deviation.

When comparing the union group with the non-union group, there was no statistical significance between age, gender, smoking status, diabetes mellitus or mechanism of injury (Table 2). There was also no statistical significance between the distribution of limbs by Gustilo-Anderson grade or by a MESS score, with regards to primary tibial union. The presence of arterial injury was 9 (15%) in the primary union group and 9 (23%) in the non-union group but this was not found to be statistically significant (*p* = 0.30) and post-hoc power was 6.2%.

**Table 2:** Demographics, co-morbidities, and injury severity of patient population separated by primary union vs. primary non-union.

	Primary union (*n* = 61)	Non-union (*n* = 39)	*p*-value
Average Age (years ± SD)	42.3 ± 17.4	44.1 ± 17.8	0.72^^^
Gender (%)			0.35^#^
Male	48 (79)	27 (69)	
Female	13 (21)	12 (31)	
Smoker (%)	18 (30)	17 (44)	0.20^#^
Diabetes (%)	2 (3)	3 (8)	0.38^#^
Mechanism of Injury (%)			
Motor Vehicle	20 (33)	9 (23)	0.37^#^
Motor Bicycle	20 (33)	16 (41)	0.52^#^
Pedestrian vs. Vehicle	8 (13)	7 (18)	0.57^#^
Cyclist	0 (0)	2 (5)	0.15^#^
Crush/Industrial	6 (10)	1 (3)	0.24^#^
Ballistic	1 (2)	0 (0)	1.00^#^
Fall	4 (7)	4 (10)	0.71^#^
Sport	1 (2)	0 (0)	1.00^#^
Aviation	1 (2)	0 (0)	1.00^#^
Gustilo-Anderson Grade (%)			
1	0 (0)	0 (0)	1.00^#^
2	2 (3)	4 (10)	0.21^#^
3A	6 (10)	4 (10)	1.00^#^
3B	47 (77)	26 (67)	0.36^#^
3C	6 (10)	5 (13)	0.75^#^
MESS ≥ 7 (%)	21 (34)	10 (26)	0.38^#^

SD, standard deviation.

^ Mann–Whitney *U* Test.

^#^ Fisher exact 2 × 2 test.

* *p* < 0.05 = statistically significant.

Twelve of the 100 patients undergoing limb salvage had external fixation as their definitive fracture fixation. Of these 12 patients, 3 (25%) patients went on to unite primarily, but 9 (75%) patients went onto primary non-union, and this was statistically significant (*p* = 0.01) and post-hoc power was 32.3%. While the non-union group had a higher percentage of comminuted tibial fractures, 36% vs. 30%, this was not found to be statistically significant (*p* = 0.52; Table 3).

**Table 3:** Comparing primary union and non-union for tibial fracture and orthopedic reconstruction.

	Primary union (*n* = 61)	Non-union (*n* = 39)	*p*-value
Fracture Pattern (%)			
Comminuted	18 (30)	14 (36)	0.52^#^
Segmental	13 (21)	10 (26)	0.46^#^
Other	30 (49)	15 (38)	0.31^#^
Definitive fixation Type (%)			
Ex-Fix	**3**(**5)**	**9**(**23)**	**0**.**01**^#^*
IMN	31 (51)	18 (46)	0.69^#^
ORIF	21 (34)	8 (21)	0.18^#^
IMN & ORIF	6 (10)	4 (10)	1.00^#^

IMN, intramedullary nail; ORIF, open reduction and internal fixation; Ex-Fix, external fixator.

^#^ Fisher exact 2 × 2 test.

* *p* < 0.05 = statistically significant.

The median timing from injury to soft tissue reconstruction was 5.5 days ± 7.3 (SD) in the primary limb salvage group. The median timing from definitive fixation to soft tissue reconstruction was 3.0 days ± 3.8 (SD) in the primary limb salvage group. Neither timing from injury nor definitive fixation to soft tissue reconstruction was found to be statistically significant when comparing for tibial union or soft tissue infection. The median time from injury to soft tissue reconstruction in the union group was 6.0 days ± 7.6 (SD) compared to 5.0 days ± 4.5 (SD) in the non-union group (*p* = 0.38). While median time from injury to soft tissue reconstruction in the soft tissue infection group (*n* = 17) was longer at 7.0 ± 4.1 vs. 5.0 ± 7.1 in the no soft tissue infection group (*n* = 83) this was, however, not statistically significant (*p* = 0.48) and post-hoc power was 100%.

All 100 patients in the primary limb salvage group, underwent operative debridement of their wounds within 24 h of their injury and were given prophylactic intravenous antibiotics on arrival to the hospital. Negative pressure wound therapy was applied after initial operative debridement in 98 (98%) of patients; two (2%) patients were initially dressed with betadine packing gauze after initial debridement. These two patients did not suffer a post operative soft tissue infection. The median operative debridements prior to definitive reconstruction in the soft tissue infection group was 2.0 debridements ± 2.1 (SD) in the soft tissue infection group. This is compared with 1.0 debridement ± 0.6 (SD) in the no soft tissue infection group. This was not statistically significant (*p* = 0.67).

The most common primary soft tissue reconstruction was a free flap in 56 (56%) limbs. There were two total free flap losses, making a free flap failure rate of 3.6%. When comparing union with non-union, the type of soft tissue reconstruction was not statistically significant (Table 4). In looking at free flaps alone (*n* = 56), comparing fasciocutaneous flaps with muscle flaps the rate of primary non-union was 29% for fasciocutaneous vs. 43% for muscle flaps, however, this was not statistically significant (*p* = 0.66).

**Table 4:** Soft tissue reconstruction type in patients who had limb salvage for their open tibial fracture.

Type of soft tissue reconstruction (%)	Primary union (*n* = 61)	Non-union (*n* = 39)	*p*-value
Direct closure	5 (8)	7 (18)	0.21^#^
Skin graft	9 (15)	5 (13)	1.00^#^
Local skin flap	4 (7)	2 (5)	1.00^#^
Pedicled muscle flap	4 (7)	8 (21)	0.06^#^
Gastrocnemius	*3*	*7*	
Hemi-soleus	*0*	*2*	
Tibialis anterior	*1*	*0*	
Free flap	39 (64)	17 (44)	0.06^#^
ALT/AMT	*29*	*10*	
Parascapular	*3*	*1*	
Radial forearm	*3*	*3*	
Latissimus Dorsi	*2*	*2*	
Gracilis	*1*	*0*	
Rectus Abdominis	*1*	*1*	

ALT, anterolateral thigh; AMT, anteromedial thigh.

^#^ Fisher exact 2 × 2 test.

* *p* < 0.05 = statistically significant.

The overall soft tissue complication rate in primarily salvaged limbs was 37% (*n* = 37) and these complications are summarized in Table 5. Seventeen primarily salvaged limbs (17%) had a soft tissue infection in the post-reconstruction phase as their soft tissue complication. There was statistical significance (*p* = 0.01) when comparing the rate of soft tissue infections in the primary union group (*n* = 4 of 61 limbs; 7%) with the non-union group (*n* = 13 of 39 limbs; 33%). In other words, soft tissue infection during the healing after reconstruction was associated with a significantly higher tibial non-union rate.

**Table 5:** Soft tissue complications reported in patients who had limb salvage for their open tibial fracture.

	Primary union (*n* = 61)	Non-union (*n* = 39)	*p*-value
Soft tissue complication per limb (%)	15 (25)	22 (56)	0.01^#,^*
Soft tissue infection	4 (7)	13 (33)	0.01^#,^*
Hematoma	2 (3)	2 (5)	0.64^#^
Native skin necrosis	4 (7)	2 (5)	1.00^#^
Graft loss	1 (2)	1 (3)	1.00^#^
Free flap complication	3 (5)	2 (5)	1.00^#^
Infection	*0*	*2*	
Venous compromise	*2*	*0*	
Arterial insufficiency	*1*	*0*	
Partial free flap loss	*2*	*0*	
Total free flap loss	*0*	*2*	
Muscle flap complication	1 (2)	2 (5)	0.56^#^
Partial muscle flap loss	*0*	*1*	
Total muscle flap loss	*1*	*1*	
Local flap complication	0 (0)	1 (3)	0.39^#^
Partial local flap loss	*0*	*0*	
Total local flap loss	*0*	*1*	
Secondary Amputation	0 (0)	1 (3)	0.39^#^

^#^ Fisher exact 2 × 2 test.

* *p* < 0.05 = statistically significant.

Three (3%) patients did not have their mobility status recorded. Ninety-six (96%) patients who underwent primary limb salvage were confirmed to be mobilizing on the affected limb at their final follow-up. Sixty-seven (67%) of these patients were mobilizing independently and 29 (29%) of these patients were mobilizing with a gait aid. The one (1%) patient who had a secondary amputation and was confirmed to be weightbearing with a prosthesis. Patients who ultimately achieved tibial union were more likely to be pain-free compared to those with persistent non-union, 37% vs. 25% respectively, however this was not statistically significant (*p* = 0.41; Table 6). Patients with tibial union were more likely to mobilise independently, 73% vs. 38% (*p* = 0.01), while patients with persistent non-union were more likely to need a gait aid for mobility, 50% vs. 25% (*p* = 0.02; Table 6).

**Table 6:** Mobility and pain reported by patients who had an open tibial fracture.

	Total union (*n* = 84)	Persistent non-union (*n* = 16)	Total (*n* = 100)	*p*-value
Pain reported (%)				
None	31 (37)	4 (25)	35 (35)	0.41^#^
Minor pain	37 (44)	5 (31)	42 (42)	0.42^#^
Chronic pain	12 (14)	5 (31)	17 (17)	0.14^#^
Not recorded	4 (5)	2 (13)	6 (6)	0.24^#^
Mobility (%)				
Wheelchair	0 (0)	0 (0)	0 (0)	1.00^#^
Prosthesis	0 (0)	1 (6)	1 (1)	0.16^#^
Gait aid	21(25)	8(50)	29(29)	0.02^#,^*
Independent	61(73)	6(38)	67(67)	0.01^#,^*
Not recorded	2 (2)	1 (6)	3 (3)	0.41^#^

^#^ Fisher exact 2 × 2 test.

* *p* < 0.05 = statistically significant.

## 4 Discussion

A successful outcome in primary lower limb salvage should be defined as a united pain-free extremity that allows independent mobility (5, 8). This success requires appropriate resuscitation, antibiotic administration, timely and comprehensive debridement, fracture stabilization and appropriate soft tissue reconstruction (1–8). In recent times, the concept of “fix and flap”, where bony fixation and soft tissue reconstruction occur in the same operative episode, has come into favor in many institutions (1–3, 14). Management of these patients is a logistical challenge and requires the co-ordination of multiple specialties and resources including the trauma unit, orthopedic surgery, plastic surgery, vascular surgery and intensive care as well as access to dedicated operative time (1, 2, 5). These realities, along with interhospital transfers and the critically unwell nature of multi-trauma patients, can create delays in definitive management of an open tibial fracture (3, 14, 15). In this study, time to definitive soft tissue reconstruction did not influence post operative infection or primary tibial union.

There is a relative paucity of literature showing primary and secondary tibial union rates in high-energy open injuries (1, 2, 8). A meta-analysis by Haykal et al. in 2018 (8), showed no difference in time to bony union between early and delayed reconstruction patients and reported that tibial union took approximately 12 months. In this current study, timing from injury to soft tissue reconstruction was not a statistically significant factor between the union and non-union groups. We have demonstrated an overall union rate of 84%. The primary bony union rate of 61% was also comparable to the Gopal et al. (1) rate of 66%. In our study, two factors were statistically significant between the primary union and non-union groups. Firstly, using an external fixator as definitive fracture fixation and secondly, a postoperative soft tissue infection. There was a trend showing higher non-union in high energy injuries and higher rate of infection in the delayed soft tissue reconstruction group, but these were not significant. While Gopal et al. (1) and Naique et al. (2) described a preference against definitive external fixation due to difficulties with soft tissue reconstruction and pin-site infections, they did not show a statistically significant difference in union rates. In the current cohort, definitive external fixation was generally employed in heavily comminuted and unstable tibial fractures thus a fracture pattern that may have been more likely to be denuded of periosteum and lead to non-union. While fracture pattern did not show statistical significance, it was not able to be separated for degree of comminution in this study. The association found between soft tissue infection and tibial non-union has been previously reported (2, 5, 8).

Ideal timing of soft tissue reconstruction in open tibial fractures has been extensively described (1–10, 14–18). Tibial union rates, patient mobility and pain post limb salvage are however reported less commonly and more variably (1, 2, 5, 7). The current literature reports a range from 42%–95% for mobilization (1, 2, 5, 7), however, the use of gait aids or independent mobilization are not necessarily stated. Our current study had total confirmed mobilization rate of 96% with an aid free mobilization rate of 67% and a further 29% mobilizing with a gait aid such as a crutch, walking stick or walking frame. Pain in the injured limb was infrequently reported in most studies (2, 7, 8). In this cohort, 35% of primary limb salvage patients reported no pain and a further 42% reported minor or occasional pain in their injured limb. Interestingly and importantly, despite not achieving primary tibial union, the non-union group were still able to mobilise in all but one case, although were more likely to require the use of a gait aid.

In this study, one of the main factors associated with non-union is soft tissue infection. It is often believed that early soft tissue coverage will decrease the risk of infection. However, a balance must be found between the possibility of under debridement of soft tissues where tissue viability is difficult to assess and time to demarcate is required. Negative pressure dressings have helped minimizing bacterial contamination and the introduction of antibiotic prophylaxis has also reduced the risk of infection (1–3, 19). During the study period, at our institution cefazolin 2 grams intravenously 8-hourly was given routinely for clean open fractures and piperacillin and tazobactam 4.5 grams intravenously 8-hourly was given for heavily contaminated open fractures.

Despite the major prospective collection of information in the lower limb database, a limitation in this study was the subsequent collection of late postoperative complications, mobility, and pain from clinical notes. There were inconsistent and sometimes prolonged time intervals in the post fixation radiographs, mobility documentation and pain assessments. This means that time to union and time to mobilization outcomes are likely to be overstated. The lack of Gustilo-Anderson Grade 1 and low numbers of Grade 2 and 3A patients in this study relates to the fact that these less severely injured patients were managed by orthopedics alone without plastic surgical referral. The ability to reach statistical significance in some analyses was constrained by the sample sizes.

## 5 Conclusions

Management of open tibial fractures is a logistical challenge, requiring a multidisciplinary approach and dedicated resources. Modern surgical practices are such that microvascular failure rates are low, but this is only one part of the care that is required. Success in limb salvage requires timely and adequate resuscitation, antibiotic administration, debridement, fracture stabilization, and appropriately designed and timed soft tissue reconstruction in a systemically stable patient.

Current guidelines advocate for definitive soft tissue coverage within seven days of injury. At our institution we do aim for judicious early soft tissue coverage, but not at the expense of other important steps in the care of these patients. Definitive fracture stabilization using external fixation and soft tissue infections were both factors found to be associated with lower union rates in this study. Duration from initial injury to final reconstruction was not associated with an increased rate of soft tissue complications in this study. Importantly, it was also not associated with an increase in non-union, pain or decreased mobility.

Open tibial fracture treatment can be considered a success if the patient can return to comfortable mobilization using the affected limb. This significant study demonstrates an ultimate tibial union rate of 84% and a confirmed ambulation rate of 96% after undertaking primary limb salvage at this major trauma center.

## Data Availability

The original contributions presented in the study are included in the article/Supplementary Material, further inquiries can be directed to the corresponding author.
